# Strengthening trauma and mass-casualty preparedness in Somalia: an urgent priority for emergency care systems

**DOI:** 10.1016/j.afjem.2026.100980

**Published:** 2026-05-21

**Authors:** Abdikadir Ahmed Hassan, Liibaan Abdulahi Sudi, Mohamed Daud Mohamed, Iqro Omar Ibrahim, Yakub Burhan Abdullahi

**Affiliations:** a Faculty of Health Sciences and Tropical Medicine, Somali National University, Mogadishu, Somalia; b Faculty of Health Sciences, Salaam university, Mogadishu, Somalia; c Center for Health Research and Innovation, Somali National University, Mogadishu, Somalia; d Mogadishu Institute of Health, Mogadishu, Somalia

**Keywords:** Trauma preparedness, Mass-casualty incidents, Emergency care systems, Somalia, Emergency medicine

Over one-third of mass-casualty incident victims in Mogadishu die, and one in five are already dead upon hospital arrival [1]. These deaths should not be interpreted as the inevitable consequence of conflict. Rather, they are measurable signs of failure across the emergency care continuum, including delayed scene response, limited transport capacity, inadequate triage, constrained operative capacity, and insufficient critical-care support. Therefore, trauma and mass-casualty preparedness in Somalia is a core test of whether emergency care systems can deliver timely, life-saving care [5].

The available evidence from Somalia is limited but is becoming increasingly consistent. A nationwide assessment of 131 hospitals using the WHO Hospital Emergency Unit Assessment Tool (HEAT) reported a median emergency and critical care readiness score of only 0.31 (IQR 0.22–0.46), with more than 80% of facilities falling below the basic readiness threshold [1]. These findings indicate system-wide weaknesses in time-critical care delivery and surge capacity. Long-term facility-based data reinforce this pattern. Across seven years at a Mogadishu referral hospital, 2,426 patients were hospitalized after 359 terrorism-related explosions and firearm attacks, with an inpatient mortality rate of 11.6% [4]. A retrospective analysis of 400 mass-casualty victims from 2013 to 2018 in two Mogadishu hospitals demonstrated mortality exceeding one-third, with 21.3% brought in dead and 39% dying in the operating theatre [2]. Together, these findings point to failures not only in trauma resuscitation but also in prehospital access, referral coordination, and operative surge capacity.

These patterns are directly relevant to the emergency care system agenda. The Emergency Care System Assessment framework structures system evaluation across governance and financing, data and quality improvement, scene care and transport, facility-based care, and emergency preparedness [5]. Trauma deaths during mass-casualty events rarely reflect a single missing intervention; they arise when weaknesses in transport, triage, operating capacity, intensive care, coordination, and quality oversight reinforce each other.

Importantly, the evidence suggests that improvements are possible. A WHO Mass Casualty Management course delivered to trauma hospital teams across three Somali regions in October 2023 increased mean knowledge scores from 47.9% to 78.9%, with the largest gains in operational readiness competencies, including Emergency Department clearance, documentation, and triage planning [3]. Although training alone cannot overcome structural deficits, these findings show measurable gains in terms of preparedness.

Several priorities should now be pursued by the Ministry of Health and hospital leaders, with support from international donors and partners. First, the Ministry of Health should adopt a national emergency care quality plan that includes trauma and mass-casualty preparedness as a core systems objective, with benchmarks for scene-to-facility transfer, triage performance, operative surge, and critical care escalation [5]. Second, hospital leaders and partners should institutionalize and scale Mass Casualty Management training and simulation drills nationally, using competency-based assessments [3]. Third, preparedness efforts must be matched by investments in baseline emergency capacity, including essential equipment, oxygen, workforce support, and the removal of point-of-care financial barriers [1]. Fourth, the Ministry of Health should establish a national trauma registry and injury surveillance system to support data-driven planning, prioritization, and quality improvement.

## Dissemination of results

The messages and recommendations of this correspondence may be disseminated through academic publication, professional emergency care networks, and engagement with clinicians, educators, and health policy stakeholders in Somalia and similar African settings. Dissemination through these channels may support awareness, discussion, and future action on trauma system strengthening, emergency care preparedness, and mass-casualty response. Given the policy and systems focus of the manuscript, its findings may also be relevant to ministries of health, hospital leaders, emergency care trainers, and organizations involved in emergency preparedness and health system strengthening.

## Sources of funding

The authors received no financial support for the research, authorship, and/or publication of this article.

## Ethical approval

Ethical approval was not required for this study.

## Consent to participate

Not applicable.

## Consent for publication

Not applicable.

## Availability of data and materials

No new data were generated or analyzed in this study. Therefore, data sharing is not applicable.

## CReDiT author contributions

A.A.H. conceptualized the study. L.A.S. developed the methodology, conducted the investigation, and prepared the original draft of the manuscript. L.A.S. and I.O.I. contributed to the investigation and analysis. M.D.M. and I.O.I. critically reviewed and edited the manuscript. Y.B.A. provided overall supervision and guidance throughout the study. All authors read and approved the final version of the manuscript and agree to be accountable for all aspects of the work.

## Declaration of competing interest

The authors declare that they have no known competing financial interests or personal relationships that could have influenced the work reported in this study.
